# The behavioural phenotype of Potocki-Lupski syndrome: a cross-syndrome comparison

**DOI:** 10.1186/s11689-017-9221-x

**Published:** 2018-01-10

**Authors:** Stacey Bissell, Lucy Wilde, Caroline Richards, Jo Moss, Chris Oliver

**Affiliations:** 1 Cerebra Centre for Neurodevelopmental Disorders, School of Psychology, University of Birmingham, Birmingham, B15 2TT UK; 2 Institute of Cognitive Neuroscience, University College London, Alexandra House, 17-19 Queen Square, London, WC1N 3AR UK

**Keywords:** Autism spectrum disorder, Behavioural phenotype, Challenging behaviour, Impulsivity, Potocki-Lupski syndrome, Repetitive behaviour, Self-injury, Smith-Magenis syndrome

## Abstract

**Background:**

Potocki-Lupski syndrome (PTLS) and Smith-Magenis syndrome (SMS) are related genomic disorders, as duplication 17p11.2 (associated with PTLS) is the reciprocal recombination product of the SMS microdeletion. While SMS has a relatively well-delineated behavioural phenotype, the behavioural profile in PTLS is less well defined, despite purported associations with autism spectrum disorder (ASD) and the suggestion that some behaviours may be diametric to those seen in SMS.

**Methods:**

Caregivers of individuals with PTLS (*N* = 34; *M* age = 12.43, *SD* = 6.78) completed online behavioural questionnaires, including the Challenging Behaviour Questionnaire (CBQ), the Activity Questionnaire (TAQ), the Repetitive Behaviour Questionnaire (RBQ), the Mood, Interest and Pleasure Questionnaire-Short Form (MIPQ-S) and the Social Communication Questionnaire (SCQ), which assesses behaviours associated with ASD. Individuals with PTLS were matched on age and adaptive functioning to individuals with SMS (*N* = 31; *M* age = 13.61, *SD* = 6.85) and individuals with idiopathic ASD (*N* = 33; *M* age = 12.04, *SD* = 5.85) from an existing dataset.

**Results:**

Individuals with PTLS and SMS were less impaired than those with idiopathic ASD on the communication and reciprocal social interaction subscales of the SCQ, but neither syndrome group differed from idiopathic ASD on the restricted, repetitive and stereotyped behaviours subscale. On the repetitive behaviour measure, individuals with PTLS and idiopathic ASD scored higher than individuals with SMS on the compulsive behaviour subscale. Rates of self-injury and property destruction were significantly lower in PTLS and idiopathic ASD than in SMS. No between-syndrome differences were found in relation to overactivity or mood; however, impulsivity was greater in SMS than in PTLS.

**Conclusions:**

Findings suggest some overlap in the behavioural phenotype of PTLS and features of ASD symptomatology; however, the overall profile of behaviours in PTLS appears to be divergent from both idiopathic ASD and SMS. Relative to idiopathic ASD, PTLS is not characterised by communication or social interaction deficits. However, restricted and repetitive behaviours were evident in PTLS, and these may be characterised specifically by compulsive behaviours. While several behavioural differences were identified between PTLS and SMS, there was little evidence of diametric behavioural phenotypes, particularly in relation to social behaviour.

## Background

Potocki-Lupski syndrome (PTLS) occurs in approximately 1 in 25,000 births [1] and is associated with congenital anomalies and intellectual disability (ID) [2]. PTLS is caused by genetic duplication within the 17p11.2 region, which comprises low copy repeat (LCR) gene clusters known as SMS-REPs [3, 4]. The proximal and distal SMS-REPs located on chromosome 17p11.2 are areas of genetic instability that are highly susceptible to non-allelic homologous recombination [5]. Although PTLS is most commonly associated with a ~ 3.7-Mb duplication, larger and smaller duplications have also been reported [1, 6]. Duplication of *PMP22* at 17p12 causes Charcot-Marie-Tooth disease type 1A (CMT1A) [6]. Large duplications that encompass both the PTLS region at 17p11.2 and *PMP22* have been reported, and the resulting clinical phenotype is termed Yuan-Harel-Lupski syndrome (YUHAL) [7]. PTLS is characterised by poor feeding in infancy, infantile hypotonia, cognitive impairment, cardiovascular abnormalities and speech and language impairment [1, 6, 8, 9], whereas the YUHAL phenotype is typically more severe, in relation to motor delay and onset of clinical neuropathy [7].

It has been difficult to ascertain whether there is a distinctive behavioural phenotype associated with PTLS (the subset of behaviours that occur more often in individuals with the disorder than in individuals without the disorder [10]), because it is a recently recognised syndrome [1], and the PTLS duplication cannot easily be detected using cytogenetic analysis. Clinical case reports have documented repetitive behaviours and vocalisations, anxiety, hyperactivity, attention deficits and behavioural problems in some individuals with PTLS [11–13]. Two larger scale multidisciplinary studies have been conducted to delineate the behavioural profile of PTLS [1, 14], in which repetitive behaviours, hyperactivity, anxiety, withdrawal and inattention and behaviours associated with autism spectrum disorder (ASD) were described. However, the extent to which these behaviours can be considered phenotypic is limited, due to the absence of comparison groups, which are required to establish whether behaviours are more likely in a given syndrome.

PTLS duplications are the reciprocal recombination products of the Smith-Magenis syndrome (SMS) microdeletions. The dosage-sensitive retinoic acid inducible 1 (*RAI1*) candidate gene is implicated in both PTLS and SMS [6, 15]. *RAI1* is associated with transcript regulation, although it is unclear exactly how *RAI1* deletions, duplications or mutations relate to the clinical and behavioural manifestations in these genomic disorders [16]. There is substantial overlap in the phenotype of individuals with SMS resulting from specific *RAI1* point mutations and those with SMS caused by 17p11.2 deletions [17, 18], suggesting that the core features of SMS, including most aspects of the neurobehavioural phenotype, are caused by haploinsufficiency of *RAI1*. The common clinical phenotype associated with SMS is mild to moderate ID, hearing impairments, delayed speech, dysmorphic facial features and cardiovascular and renal abnormalities, amongst many others [17, 19–21]. Its behavioural phenotype is very distinctive; high prevalence of self-injury and aggression, upper body hugging, repetitive page ‘lick and flip’, mouthing of hands or objects, insertion of fingers and objects into other body orifices, removal of fingernails and sleep difficulties due to an inverted circadian rhythm of melatonin are well documented [4, 17, 19, 22, 23]. Some literature suggest larger deletions can result in greater phenotypic severity in relation to the behavioural phenotype [17, 24]; however, this has not been substantiated in other clinical reports [20]. Individuals with *RAI1* point mutations as opposed to 17p11.2 deletions are less likely to present with short stature, cardiac anomalies and hearing problems and are more likely to exhibit certain characteristics relating to food intake, obesity, self-hugging and skin picking, for example [17, 18]. Therefore, the role of *RAI1* in these phenotypic characteristics may depend on whether haploinsufficiency of *RAI1* is caused by a mutation versus a deletion of this gene (and other genes in the 17p11.2 region). Interestingly, *RAI1* overexpression in PTLS is also believed to disrupt circadian gene expression resulting in sleep difficulties, which suggests *RAI1* is implicated in the phenotype of both SMS and PTLS in this respect [25].

Given the genetic relationship between PTLS and SMS, it has been suggested that traits may be mirrored or diametrically opposed in these two syndromes [26–28]. ‘Mirror traits’ are described by Lupski [27] as aspects of a syndrome that are diametric or opposing to those seen in its related or sister disorder (e.g. duplication and deletion recombination reciprocal disorders) and is the term that will be adopted here. Supporting evidence of diametric phenotypes comes from mouse models of PTLS and SMS, in which mice with the common PTLS duplication display mirror traits (hyperactivity, impaired contextual fear conditioning and intact learning), in comparison to mice with the common SMS deletion (hypoactivity, an intact fear conditioning response and marked learning deficits) [29–31], and from reports of diametric levels of sociability between individuals with PTLS and SMS (see [26] for a review). Compared to PTLS, relatively good social skills are reported in the SMS literature, such as maintained eye contact, high levels of social engagement and a keen sense of humour [32], which is offset by a significant preference for adult attention as opposed to peer attention [33, 34].

Comparisons between PTLS and SMS may be informative on two levels. Clinically, contrasts with a well-delineated syndrome provide families and professionals with valuable context to anchor the severity, topography and prevalence of behaviours seen in the individuals they care for with PTLS. From a theoretical perspective, cross-syndrome comparisons between PTLS and SMS may help to elucidate genotype-phenotype correlations between copy number variation at 17p11.2 and behaviour. In other genetic syndromes that share genetic anomalies within the same region, it has been possible to make such behavioural cross-syndrome comparisons. Angelman syndrome (AS) and Prader-Willi syndrome (PWS) are sister disorders, related to maternal and paternal genomic imprinting defects, respectively, within the same 15q11–q13 region [35–37]. Despite this shared genetic basis, distinctive behavioural phenotypes, or mirror traits, are described in AS (frequent laughing and smiling, repetitive stereotypies, overactivity) and PWS (food-seeking behaviours, temper outbursts, impulsivity) [35, 36, 38, 39].

However, evidence of mirrored behavioural traits is tentative in PTLS, given that atypical sociability may not necessarily be phenotypic of the syndrome. It is particularly difficult to identify ASD behaviours in individuals with genetic syndromes associated with ID, as there may be qualitative differences in ASD phenomenology between individuals with a genetic syndrome and individuals with idiopathic ASD [40]. Although ASD behaviours have been identified in several individuals with PTLS, studies reporting this vary in terms of whether they assess behaviours using standardised measures [41] or whether behaviours are described simply as ‘features’ characteristic of ASD [12]. Furthermore, there are a number of PTLS case reports, which explicitly state an absence of ASD behaviours [42], and literature that also suggests individuals with PTLS can show relative strengths in social interaction (e.g. joint attention, appropriate play), which would not be indicative of the behavioural profile seen in idiopathic ASD [14].

To establish whether atypical social behaviours are associated with PTLS, it is also of clinical interest to compare social behaviours to an idiopathic ASD group. Therefore, the aim of the present study was to further delineate the behavioural phenotype of PTLS via comparisons to two disorders: SMS and idiopathic ASD.

## Methods

### Recruitment and participants

Families were recruited primarily through two support group organisations: Unique based in the UK and the Potocki-Lupski Syndrome Outreach Foundation based in the USA. All participants had received a diagnosis of PTLS from a paediatrician, clinical geneticist, general practitioner or neurologist. Data from five participants could not be analysed, as fewer than 75% of questionnaire items had been completed and three participants with an additional diagnosis of CMT1A were removed from analysis.

Therefore, 34 individuals with PTLS were matched, first according to level of adaptive ability as determined by the self-help subscale of the Wessex Scale [43] (+/− 2 points) and then by age (+/− 2 years), to an idiopathic ASD group and SMS group, derived from a database of participants who have participated in previous research and consented for their data to be used in future research. Individuals with ASD had a confirmed diagnosis from a relevant professional, paediatrician, general practitioner, psychiatrist, clinical psychologist, or educational psychologist, and the SCQ was used as an additional screening measure. One individual from the idiopathic ASD group and three individuals from the SMS group could not be matched within two points and 2 years to four individuals with PTLS, hence the unequal group numbers. The data in Table 1 indicate that the groups were relatively well matched according to age (*H*(2) = 1.153, *p* = .562) and level of ability (*H*(2) = .909, *p* = .635), as *p* > .50 is arguably an appropriate value to assume groups do not differ [44].

**Table 1:** Demographic information of each syndrome group and associated cross-syndrome comparisons and post hoc tests

Variables	Syndrome group			Cross-syndrome comparison	
	PTLS (*N* = 34)	SMS (*N* = 31)	ASD (*N* = 33)	*p* value	Post hoc test
Mean age^a^, years (SD)	12.43 (6.78)	13.61 (6.85)	12.04 (5.85)	.562	
% male (*N*)	55.88 (19)	45.16 (14)	87.88 (29)	.001	ASD > SMS, PTLS
Mean self-help score^a, b^ (SD)	7.18 (1.47)	7.06 (1.34)	7.39 (1.43)	.635	
% fully mobile^b^ (*N*)	79.41 (27)	77.42 (24)	90.91 (30)	.369	
% normal vision (*N*)	82.35 (28)	70.97 (22)	100.00 (33)	.005	ASD > SMS
% normal hearing (*N*)	91.18 (31)	64.52 (20)	100.00 (33)	< .001	ASD > SMS
% verbal/partly verbal^c^ (*N*)	85.29 (29)	87.10 (27)	87.88 (29)	.950	

^a^Continuous non-normally distributed data; therefore, Kruskal-Wallis tests were conducted

^b^Data derived from the Wessex Behaviour Schedule

^c^Data derived from item 1 of the Wessex Behaviour Schedule—‘Is the person you care for verbal? (more than 30 signs/words in their vocabulary)’

There were more males in the idiopathic ASD group compared to the SMS group (*χ*^2^(1) = 13.231, *p* < .001) and the PTLS group (*χ*^2^(1) = 8.439, *p* = .004). There were also more individuals in the ASD group with normal vision (*χ*^2^(1) = 11.148, *p* = .001) and normal hearing (*χ*^2^(1) = 13.075, *p* < .001), compared to the SMS group.

### Procedure

Parents and caregivers of children and adults with PTLS were invited via Unique and the Potocki-Lupski Syndrome Outreach Foundation to complete an online survey, created using LimeSurvey 2.00+ software. The online survey included an information sheet, consent forms and questionnaire measures.

### Measures

#### Demographic questionnaire

Parents and caregivers reported on the gender, age, verbal capacity, mobility and diagnosis of PTLS (such as date of diagnosis and whether an additional diagnosis of CMT1A has been made).

#### Wessex Behaviour Schedule [43]

This schedule measures the degree of ability in individuals with ID. Only the ‘incapacities’ and ‘speech’ scales were included in the present study, which measure aspects such as mobility, self-help capacity, vision and hearing. Items are rated on a three-point scale from 1 (severe impairment) to 3 (no impairment). Inter-rater reliability of these subscales in the original Wessex Behaviour Schedule ranged from 78% (self-help skills, literacy) to 92% (mobility) [43].

#### Social Communication Questionnaire (SCQ [45])

Higher scores on this 40-item measure denote greater ASD symptomology, with a cut-off score of 15 indicative of ASD. Thirty-six of the items are subdivided into three domains, reciprocal social interaction, communication and restricted, repetitive and stereotyped behaviours. Parents and caregivers are asked to indicate the occurrence of behaviours over the person’s lifetime (e.g. ‘Has she/he ever seemed to be unusually interested in the sight, feel, sound, taste or smell of things or people?’) and also between the ages of four and five. This measure has good diagnostic validity, with sensitivity and specificity values of .92 and .62, respectively, when a cut-off score of 15 for ASD is utilised [46].

#### Repetitive Behaviour Questionnaire (RBQ [47])

Informants indicate the occurrence of 19 observable, operationally defined behaviours (e.g. hoarding, repetitive questions, lining up or arranging objects), which can be grouped according to five domains of repetitive behaviour: stereotyped behaviour, compulsive behaviour, insistence on sameness, restricted preferences and repetitive speech. Restricted preferences and repetitive speech are not calculated for individuals with limited verbal ability. Correlation coefficient values of .46–.80 for inter-rater reliability and .61–.93 for test-retest reliability have been attained [48].

#### Challenging Behaviour Questionnaire (CBQ [49])

The CBQ determines the presence of self-injurious, aggressive, destructive and stereotyped behaviours, as well as the topography, duration, consequence and frequency of self-injurious behaviours in the last month, if applicable (e.g. rubs or scratches self). Moderate to very strong kappa coefficients [50] for the CBQ have been reported in relation to inter-rater reliability (.60–.92) [49].

#### Mood, Interest and Pleasure Questionnaire-Short Form (MIPQ-S [51])

The MIPQ-S is a 12-item measure; six items correspond to a ‘mood’ subscale (e.g. ‘In the last two weeks, do you think the facial expression of the person looked flat …’) and six items correspond to an ‘interest and pleasure’ subscale (e.g. ‘In the last two weeks, did the person seem to have been enjoying life …’). The MIPQ-S has good psychometric properties in relation to inter-rater and test-retest reliability, with correlation coefficient values of .85 and .97, respectively [52].

#### The Activity Questionnaire (TAQ [53])

This measure is comprised of three subscales: impulsivity, overactivity and impulsive speech. Impulsive speech is not calculated for non-verbal individuals. Respondents are asked to rate the frequency of 18 behaviours (e.g. ‘Does the person want things immediately?’) according to a five-point scale ranging from 0 (never/almost never) to 4 (always/almost all of the time). Mean inter-rater reliability and test-retest reliability correlation coefficient values of .56 and .75, respectively, have been reported [54].

### Data analysis

Data were analysed using Statistical Package for Social Sciences (SPSS) 20.0 software. Normality was assessed via Shapiro-Wilk tests, analysis of skewness and kurtosis and scrutiny of distribution plots for the identification of outliers. Levene’s test was used to determine homogeneity of variance when applicable. Chi-square tests were employed to compare categorical data between syndrome groups, and parametric one-way analyses of covariance or non-parametric Kruskal-Wallis tests were conducted to compare continuous data between groups. Significant group differences (set at *p* < .01 to control for multiple group comparisons) were followed up with the appropriate categorical (one-way chi-square test), parametric (independent *t* test) or non-parametric (Mann-Whitney test) post hoc analyses (*p* < .01).

## Results

### Prevalence of ASD behaviours and repetitive behaviours

The percentage of individuals from each syndrome group who scored above the clinical cut-off score for ASD is presented in Table 2. As expected, more individuals with idiopathic ASD met the clinical cut-off score for ASD than the PTLS group (*χ*^2^(1) = 14.188, *p* < .001) or the SMS group (*χ*^2^(1) = 12.443, *p* < .001).

**Table 2:** Percentage of individuals meeting the SCQ cut-off score for ASD (≥ 15)

SCQ cut-off scores	Syndrome group			Cross-syndrome comparison	
	PTLS (*N* = 34)	SMS (*N* = 28)^a^	ASD (*N* = 33)	*p* value	Post hoc test
Score ≥ 15 (%) (*N*)	64.71 (22)	67.86 (19)	100.00 (33)	.001	ASD > PTLS, SMS

^a^SCQ data were incomplete for three individuals with SMS, and therefore, SCQ total scores could not be calculated

As shown in Table 3, individuals with idiopathic ASD attained higher scores than the other groups on the reciprocal social interaction subscale (SMS *U*(1) = 208.50, *Z* = − 3.679, *p* < .001; PTLS *U*(1) = 205.50, *Z* = − 4.470, *p* < .001) and communication subscale (SMS *U*(1) = 212.00, *Z* = − 3.628, *p* < .001; PTLS *U*(1) = 305.00, *Z* = − 3.221, *p* = .001) of the SCQ. These differences remained even when verbal items (items 2–7) were removed from analysis, indicating such differences were not due to verbal ability affecting scores on the communication subscale. Individuals with PTLS did not differ significantly from those with SMS on either of these subscales (reciprocal social interaction *U*(1) = 337.50, *Z* = − 1.968, *p* = .049; communication *U*(1) = 403.00, *Z* = − 0.037, *p* = .300). There were no statistically significant differences between groups on the restricted, repetitive and stereotyped behaviour subscale of the SCQ (*H*(2) = 5.259, *p* = .072).

**Table 3:** Median subscale scores of each behavioural measure and associated cross-syndrome comparisons and post hoc tests

	Syndrome group median scores (interquartile range)^c^			Cross-syndrome comparison	
Measures	PTLS	SMS	ASD	*p* value	Post hoc test
Social Communication Questionnaire					
Reciprocal social interaction	4.00 (2.00–7.25)	7.00 (4.00–8.75)	11.00 (8.00–13.00)	< .001	ASD > PTLS, SMS
Communication^a^	6.75 (4.66–8.03)	6.00 (3.79–8.00)	9.00 (7.00–11.00)	< .001	ASD > PTLS, SMS
Restricted, repetitive and stereotyped behaviours	5.00 (3.00–7.00)	5.00 (3.00–6.00)	6.00 (4.00–7.50)	.072	
Repetitive Behaviour Questionnaire					
Stereotyped behaviour	4.00 (0.00–8.00)	8.00 (3.00–11.00)	6.00 (2.50–11.50)	.034	
Compulsive behaviour	12.00 (2.75–15.25)	2.00 (0.00–5.00)	8.00 (3.00–18.00)	< .001	PTLS, ASD > SMS
Insistence on sameness	2.00 (0.00–7.00)	3.00 (1.00–4.00)	5.00 (2.25–8.00)	.014	
Restricted preferences^a^	5.00 (3.50–8.00)	5.00 (4.00–8.00)	4.00 (2.00–8.50)	.814	
Repetitive speech^a^	4.00 (2.00–8.00)	6.00 (4.00–9.00)	6.00 (2.00–9.50)	.334	
Challenging Behaviour Questionnaire					
Self-injury severity score	6.50 (4.25–9.00)	7.00 (5.00–9.00)	7.00 (5.00–8.00)	.894	
Mood, Interest and Pleasure Questionnaire-Short Form					
Mood	21.00 (19.00–23.00)	20.00 (17.00–22.00)	18.00 (17.00–21.00)	.092	
Interest and pleasure^b^	17.26 (15.65–18.87)	16.25 (14.47–18.02)	13.71 (12.05–15.38)	.008	PTLS > ASD
The Activity Questionnaire					
Impulsivity	13.00 (7.75–19.00)	20.00 (16.00–24.00)	19.00 (11.50–21.00)	.002	SMS > PTLS
Overactivity	10.00 (5.50–20.50)	16.00 (9.00–27.00)	20.00 (8.00–29.00)	.090	
Impulsive speech^a^	4.00 (1.00–5.00)	5.00 (2.00–9.00)	5.00 (3.00–9.50)	.057	

^a^Subscales differ depending on verbal ability. Scores are prorated for non-verbal individuals (SCQ communication) or are not scored for non-verbal individuals, and therefore, the group *n* differs for these subscales (RBQ restricted preferences, RBQ repetitive speech, TAQ impulsive speech)

^b^Normally distributed data; therefore, parametric analysis of variance (ANOVA) was conducted (means and 95% confidence intervals reported)

^c^Interquartile range based on weighted average at 25 and 75 percentiles

There were no overall between-group differences identified on the majority of the RBQ subscales (stereotyped behaviour *H*(2) = 6.775, *p* = .034; insistence on sameness *H*(2) = 8.472, *p* = .014; restricted preferences *H*(2) = .413, *p* = .814; repetitive speech *H*(2) = 2.191, *p* = .334), which was due to the utilisation of a more stringent adjusted *p* value for some of the subscales (e.g. stereotyped behaviour and insistence on sameness). However, a marked between-group difference was found for compulsive behaviour (*H*(2) = 16.613, *p* < .001). On this subscale, individuals with PTLS showed higher levels of compulsive behaviour than individuals with SMS (*U*(1) = 263.50, *Z* = − 3.481, *p* = .001), as did individuals with idiopathic ASD (*U*(1) = 243.50, *Z* = − 3.616, *p* < .001). No significant difference in compulsive behaviour was found between individuals with PTLS and individuals with ASD however (*U*(1) = 552.50, *Z* = − .107, *p* = .915).

### Self-injury, aggression, mood, interest and pleasure, overactivity and impulsivity

Groups differed on a number of specific CBQ items as shown in Table 4. Individuals with SMS were more likely to display self-injurious behaviour than individuals with PTLS (*χ*^2^(1) = 27.793, *p* < .001) or individuals with idiopathic ASD (*χ*^2^(1) = 20.780, *p* < .001). Scores of individuals with PTLS were not significantly different from those with idiopathic ASD (*χ*^2^(1) = .740, *p* = .390). Individuals with SMS were also more likely to show destruction of property than individuals with PTLS (*χ*^2^(1) = 18.841, *p* < .001) or individuals with idiopathic ASD (*χ*^2^(1) = 7.458, *p* = .006). Again, individuals with PTLS did not differ from individuals with idiopathic ASD (*χ*^2^(1) = 3.003, *p* = .082). However, groups did not differ on rates of physical aggression (*χ*^2^(2) = 2.269, *p* = .322) and did not differ on severity of self-injurious behaviour (*H*(2) = .223, *p* = .894), according to the CBQ.

**Table 4:** Prevalence of challenging behaviour within syndrome groups and associated cross-syndrome comparisons and post hoc tests

	Syndrome group			Cross-syndrome comparison	
% showing behaviour^a^	PTLS (*N* = 34)	SMS (*N* = 31)	ASD (*N* = 33)	*p* value	Post hoc test
Self-injury (*N*)	29.41 (10)	93.55 (29)	39.39 (13)	< .001	SMS > PTLS, ASD
Physical aggression (*N*)	50.00 (17)	67.74 (21)	62.50^b^ (20)	.322	
Property destruction (*N*)	23.53 (8)	77.42 (24)	43.75^b^ (14)	< .001	SMS > PTLS, ASD

^a^Stereotyped behaviour prevalence from the CBQ not included as repetitive behaviours are discussed as part of the RBQ

^b^Data missing for one individual with ASD. Percentages calculated based on 32 participants

Median subscale scores attained by each syndrome group on behavioural measures relating to mood, interest and pleasure, overactivity and impulsivity are also presented in Table 3 (except for the interest and pleasure subscale of the MIPQ-S, for which the mean group scores and 95% confidence intervals are reported). On the MIPQ-S, individuals with PTLS showed higher levels of interest and pleasure compared to individuals with idiopathic ASD (*t*(65) = − 3.125, *p* = .003) but not individuals with SMS (*t*(63) = − .870, *p* = .388). Individuals with SMS did not differ significantly from those with idiopathic ASD (*t*(62) = 2.128, *p* = .037). All three groups did not differ on the mood subscale (*H*(2) = 4.775, *p* = .092). Groups also did not differ on the overactivity (*H*(2) = 4.807, *p* = .090) or impulsive speech (*H*(2) = 5.747, *p* = .057) subscales of the TAQ. Individuals with SMS were however found to be more impulsive on this measure than individuals with PTLS (*U*(1) = 256.50, *Z* = − 3.573, *p* < .001) but not individuals with idiopathic ASD (*U*(1) = 363.50, *Z* = − 1.998, *p* = .046). Individuals with PTLS did not differ from those with idiopathic ASD (*U*(1) = 444.00, *Z* = − 1.470, *p* = .142).

## Discussion

This study aimed to delineate the behavioural profile of PTLS via comparisons with age- and ability-matched SMS and idiopathic ASD comparison groups. Selection of these comparison groups was motivated by the existing PTLS literature, which suggests (1) variable rates of ASD behaviours are reported in individuals with PTLS [1, 14] and (2) aspects of the behavioural profiles of PTLS and SMS may be diametric mirror traits [28], as the PTLS duplication is the reciprocal recombination product of the SMS microdeletion.

On the ASD symptomatology measure, individuals with idiopathic ASD showed greater impairment on the reciprocal social interaction and communication subscales than individuals with PTLS or SMS, but there were no group differences on the restricted, repetitive and stereotyped behaviour subscale. Therefore, the profile of ASD behaviours in PTLS may not be characterised by social interaction and communication deficits but, as in SMS [17, 22, 32], is more likely to be characterised by restricted, repetitive and stereotyped behaviours. Variability in the prevalence of ASD behaviours in the literature [1, 14, 42, 55] may be explained in part by the commonality of speech and language deficits in PTLS, as opposed to specific social deficits associated with ASD per se, or that generally, ASD behaviours in individuals with genetic syndromes are more variable than in individuals with idiopathic ASD [48, 56].

Our preliminary analyses revealed that this uneven profile of ASD behaviours may be characterised by compulsive repetitive behaviours in PTLS. This is in contrast to the profile of repetitive behaviours in SMS, which are not typically defined by compulsive behaviours [48] but are instead characterised by repetitive topographies of behaviour such as preference for routine [21, 32, 57], self-hugging [17, 22, 58] and a restricted preference for adult attention [22, 32–34, 48, 59]. Therefore, further investigation is required to delineate the profile of repetitive behaviours in individuals with PTLS.

Challenging behaviours, especially self-injury and property destruction, did not appear to be as prominent in PTLS as they are in SMS. Despite this, a substantial proportion of individuals with PTLS in our study still exhibited self-injury (29%) and property destruction (24%). Individuals with SMS also showed greater impulsivity compared to individuals with PTLS and idiopathic ASD. Groups did not differ on scores attained from the overactivity subscale however. This is not surprising, given that overactivity has been reported in both PTLS and SMS populations [1, 14, 17], and behaviours associated with attention deficit hyperactivity disorder (ADHD) are common within both syndromes [4, 60, 61].

Overall, the findings of the current study suggest that the broad profiles of behaviour in PTLS and SMS do not appear to be diametrically opposed. Although challenging behaviour was less frequent in PTLS than in SMS, it did still occur. Aggression in PTLS did not differ significantly from SMS and was not reported at a lower rate than the ASD contrast group. Similarly, while it has been proposed that social functioning may be a mirror trait in PTLS and SMS [26], there was no evidence for this in the current study. Instead, the evidence here seems to suggest the behavioural phenotypes in PTLS and SMS are distinct [28].

However, one finding that is of note in the context of mirror traits [27] is the elevated compulsivity in PTLS. Compulsive behaviour (characterised by over control of behaviour) can be conceptualised at one end of a spectrum of behavioural control and impulsivity (characterised by lack of behavioural control) at the opposite end. As SMS is associated with elevated impulsivity [58, 62], as also evidenced in the current study, it is possible that mirror traits in SMS and PTLS exist in relation to impulsivity and compulsivity. This suggestion should be treated with caution, as scores for compulsive behaviour were obtained from a measure of repetitive behaviour in individuals with ID, but measuring compulsivity in PTLS does warrant further investigation.

Despite a number of interesting findings, there are limitations in the present study. As the PTLS group was recruited internationally, direct cognitive assessments of ID, adaptive functioning or ASD behaviours could not be conducted. This study instead relied on informant-based measures of behaviour, utilised a screening measure for ASD (the SCQ [45]) rather than the ‘gold standard’ Autism Diagnostic Observation Schedule assessment [63] and used a proxy measure of self-help abilities (the Wessex Scale [43]). Without analysing the cytogenetic information of individuals in the PTLS and SMS groups, uncommon smaller or larger chromosomal aberrations may also have been present. To investigate mirror trait associations reliably, individuals would need to be matched according to the size of their chromosomal duplications and deletions. There were also fewer individuals in the SMS group with normal hearing and vision compared to the ASD group. To investigate whether this may have contributed to the differences identified, individuals with poor hearing and vision were removed from analyses. Consequently, the difference between the SMS and PTLS groups in relation to impulsivity and rates of property destruction between the SMS and ASD groups became non-significant. However, the effect size for the property destruction analysis remained the same (medium) when individuals with poor hearing and vision were removed, suggesting that these differences may have become non-significant because of loss of power as a result of smaller sample sizes, as opposed to differences in hearing and vision driving the significant outcomes. In contrast, the impulsivity effect size reduced from intermediate to small, suggesting that differences in vision and hearing may have contributed to the significant result found. Future studies would benefit from ascertaining larger samples to enable matching on these characteristics, in addition to age and level of ability.

It is also of note that individuals were not matched according to gender. Given our modest sample sizes, we prioritised matching according to age and estimates of ability rather than gender. The present study therefore enables profiles of ASD characteristics in PTLS and SMS to be contrasted to a profile of ASD characteristics that is representative of the idiopathic ASD population in which a disproportionate male to female ratio exists. Our study does not however enable us to compare the profile of ASD characteristics in PTLS and SMS to individuals with idiopathic ASD who are matched according to gender. This is a clear limitation, given that behavioural and neuroanatomical gender differences are prominently reported in the idiopathic ASD literature (e.g. reduced gyrification of the ventromedial/orbitofrontal prefrontal cortex in males with ASD compared to females with ASD relating to between-group differences in social functioning [64] and greater presentation of externalising and internalising behaviours in males and females diagnosed with ASD, respectively [65]). In future research, when larger sample sizes allow for a more comprehensive matching strategy to be utilised, or when gender can be included as a covariate in analyses, gender differences relating to social behaviour should be explored more thoroughly.

However, the clinical utility of this study’s findings should not be underestimated. PTLS is a rare and recently recognised syndrome [1]. The current study has identified likely difficulties around repetitive behaviour, which may have implications for behaviour management in this syndrome. For example, some behavioural management and intervention programmes for ASD, which specifically target social deficits (e.g. Reciprocal Imitation Training [66]), may not be wholly appropriate for individuals with PTLS. Therefore, the clinical implications of delineating the behavioural phenotype of PTLS are of particular benefit to caregivers and professionals.

## Conclusions

Both individuals with PTLS and SMS showed less impairment on SCQ subscales measuring reciprocal social interaction and communication, which suggests the profile of ASD behaviours in these syndrome groups does not match the ‘typical’ ASD behaviour profile in idiopathic ASD. Individuals with PTLS or idiopathic ASD showed more compulsive behaviour than those with SMS. Finally, along with individuals with idiopathic ASD, individuals with PTLS were rated as less impulsive and as showing less self-injury and property destruction by their caregivers than individuals with SMS. The profile of ASD behaviours and repetitive behaviours in PTLS therefore requires further exploration. Little evidence for mirror traits in PTLS and SMS emerged in the present study, although a possible compulsivity/impulsivity mirror trait warrants further investigation.

## Abbreviations

ADHD: Attention deficit hyperactivity disorder
AS: Angelman syndrome
ASD: Autism spectrum disorder
CBQ: Challenging Behaviour Questionnaire
CMT1A: Charcot-Marie-Tooth disease type 1A
ID: Intellectual disability
LCR: Low copy repeat
MIPQ-S: Mood, Interest and Pleasure Questionnaire-Short Form
PWS: Prader-Willi syndrome
RAI1: Retinoic acid inducible 1
RBQ: Repetitive Behaviour Questionnaire
SCQ: Social Communication Questionnaire
SMS: Smith-Magenis syndrome
SPSS: Statistical Package for Social Sciences
TAQ: The Activity Questionnaire
YUHAL: Yuan-Harel-Lupski syndrome
